# Clinical signs, prevalence, and hematobiochemical profiles associated with *Anaplasma* infections in sheep of North Iraq

**DOI:** 10.14202/vetworld.2020.1524-1527

**Published:** 2020-08-08

**Authors:** Donea Abdulrazak Abdullah, Fawwaz Fadhil Ali, Afrah Younis Jasim, Shola David Ola-Fadunsin, Fufa Ido Gimba, Moeena Sadeq Ali

**Affiliations:** 1 Department of Animal Production Techniques, Northern Technical University, Mosul, Iraq; 2 Department of Veterinary Parasitology and Entomology, Faculty of Veterinary Medicine, University of Ilorin, PMB 1515 Ilorin, Kwara State, Nigeria; 3 Avian Influenza Control Project Animal Health Component Desk office, Taraba State Ministry of Agriculture and Natural Resources Jalingo, Taraba State, Nigeria

**Keywords:** *Anaplasma* species, biochemical, hematological, prevalence, sheep

## Abstract

**Background and Aim:**

*Anaplasma* infection is a worldwide prevalent condition that causes significant economic losses in affected flocks. This study was conducted to determine the prevalence and clinical signs associated with ovine anaplasmosis as well as the hematological and biochemical changes associated with the disease in natural infection in North Iraq.

**Materials and Methods:**

A total of 420 sheep were appropriately examined, and the clinical signs were documented accordingly. Blood samples were collected and subjected to parasitological, hematological, and biochemical analyses.

**Results:**

*Anaplasma*-infected sheep displayed the following clinical signs: Paleness of the mucous membrane, bloody diarrhea, emaciation, pyrexia, jaundice, nasal discharge, coughing, loss of wool, nervous signs, hemoglobinuria, and lacrimation. The prevalence of *Anaplasma* infection was 66.19%, and female sheep were significantly (p<0.05) more infected than male sheep. The hematological and biochemical parameters were significantly different between *Anaplasma*-positive and *Anaplasma*-negative sheep.

**Conclusion:**

*Anaplasma* infection among sheep is a significant concern in North Iraq considering its prevalence, clinical signs, and hematological and biochemical findings, which entirely causes significant debilitating effects on sheep productivity. It is important to pay more attention toward managing tick infestation among sheep to reduce the occurrence of this rickettsial disease for a more robust livestock sector of the Iraqi economy.

## Introduction

Species of *Anaplasma* are very small rickettsial organisms belonging to the family Anaplasmataceae. They are obligate parasites found within the cells and cause animal and human anaplasmosis, a disease with a widespread distribution in the world, and are transmitted by ticks (biologically), by biting flies (mechanically), especially tabanids, and by fomites contaminated with blood [1-3].

Ovine anaplasmosis is primarily caused by *Anaplasma ovis* and *Anaplasma marginale*; it is also caused by *Anaplasma phagocytophilum* [1,4,5]. *A. ovis* is intraerythrocytic and its infection is generally subclinical; occasionally, it can be severe manifesting symptoms such as depression, anorexia, progressive hemolytic anemia, hemoglobinuria, and fever [4,6,7]. *A. marginale* is associated with acute anaplasmosis that is characterized by progressive hemolytic anemia associated with pyrexia, loss of weight, weakness, anorexia, constipation, dehydration, jaundice, labored breathing, depression, abortion, reduced milk production, and sometimes death of the infected animal [1,3]. *A. phagocytophilum* infects the neutrophil granulocytes and causes tick-borne fever in sheep, with the most common symptoms comprising high fever, loss of appetite, dullness, and decreased milk yield [1,8].

During the acute stage of the disease, a diagnosis in the individual animal is generally made on the basis of clinical signs, presence of the organism in the microscopic examination of Giemsa-stained blood smears, and hematological evidence of infection. This method is relatively inexpensive and requires less time [4].

Iraq has a large population of small ruminants (especially sheep), estimated at 9.3 million heads as of 2010, representing approximately 0.5% of the world’s sheep population. Sheep produces considerable amounts of milk, meat, wool, and skin [9] and provides a means of livelihood and employment for some of the Iraqi population. Agriculture and animal husbandry are the primary activities of the people in Nineveh, North Iraq, and anaplasmosis comprises one of the major veterinary health problems in Iraq.

Despite the importance of sheep to the economy and livelihood of the people of Iraq, and the negative implications of anaplasmosis, there is a lack of information on the clinical signs and hematological and biochemical changes associated with *Anaplasma* infection in sheep in Iraq.

Therefore, this was conducted to bridge this intellectual gap by investigating the prevalence and clinical signs associated with ovine anaplasmosis, as well as the hematological and biochemical changes connected with the disease in North Iraq, which causes economic losses due to its association with sheep production and reproduction.

## Materials and Methods

### Ethical approval

All applicable international, national, and/or institutional guidelines for the collection of blood samples from sheep were appropriately followed.

### Study design and sample collection

A total of 420 sheep from the northern part of Iraq were randomly examined between December 2018 and June 2019. The sheep used in this study were appropriately examined, and the clinical signs observed in each animal were properly recorded. After the examination, approximately 10 ml of blood was collected from each sheep through jugular venipuncture. The blood samples were collected into two different well-labeled sample bottles (one containing and the other not containing ethylenediaminetetraacetic acid). The samples were placed in a cool box and transported to the Parasitology and Clinical Pathology Laboratories of the Department of Animal Production Techniques, Northern Technical University, Mosul, Iraq.

### Parasitological analysis

Thin blood films were prepared from non-coagulated blood using standard protocols as documented by Taylor *et al*. [1] with few modifications. A drop of blood was placed on one end of a clean, grease-free glass slide and made into a thin film using a spreader (a clean glass slide). The prepared thin films were then air-dried, fixed in methanol for 5 min, and stained in freshly prepared 10% Giemsa stain for approximately 25 min. Next, the stained films were rinsed in buffered water and allowed to dry. The smears were examined using an Olympus microscope (Germany) at 1000× (oil immersion) for the presence and identification of *Anaplasma* parasites, as described by Taylor *et al*. [1].

### Hematological and biochemical analyses

Hematological parameters such as red blood cell (RBC) count, packed cell volume (PCV), hemoglobin concentration, and white blood cell (WBC) count were estimated from the non-coagulated blood samples as described earlier [10]. Erythrocyte sedimentation rate (ESR) was determined using the Westergren technique as described previously [11]. Serum extracted from the coagulated blood samples was subjected to biochemical analysis for estimating the levels of total bilirubin, total blood urea nitrogen (BUN), total protein, albumin, globulin, glucose, and calcium using standard procedures, as described by Thrall *et al*. [11].

### Statistical analysis

The data obtained in this study were first entered into Microsoft Office Excel 2012 and then used to determine the percentages and perform calculations. Chi-square test was used to determine the relationship between sex and the presence or absence of *Anaplasma* species. The hematological and biochemical parameters of *Anaplasma*-positive and -negative sheep were analysed using Student’s t-test. Statistical analysis was conducted using SPSS version 22.0 (SPSS Inc., Chicago, Illinois). The significance level was set at either p<0.01 or p<0.05, as required.

## Results

Sheep that tested positive for *Anaplasma* infection showed the following clinical signs: Paleness of the mucous membrane, bloody diarrhea, emaciation, pyrexia, jaundice, nasal discharge, coughing, loss of wool, nervous signs, hemoglobinuria, and lacrimation.

Of the 420 sheep sampled in this study, 278 were positive for *Anaplasma* species, representing 66.19% (95% confidence interval = 61.54-70.55). Female sheep exhibited 6.26 times higher infection tendency than male sheep, with the difference being statistically significant (p<0.01) (Table-1).

**Table-1:** Prevalence (%) of ovine *Anaplasma* infection between sexes in North Iraq.

Sex	Number	Number infected (%)	OR	95% CI	p-value
Female	284	226 (79.58)	6.26	4.00-9.88	<0.01^‡^
Male^†^	136	52 (38.24)	1.00		

† Reference category,

‡ Significant (p<0.05). OR=Odds ratio, CI=Confidence interval

*Anaplasma*-positive sheep had significantly lower levels of mean hemoglobin concentration, mean PCV, and mean RBC count than *Anaplasma*-negative sheep, whereas their mean WBC count and the mean ESR were significantly higher than those of *Anaplasma*-negative sheep (Table-2).

**Table-2:** Differences in the mean hematological parameters between *Anaplasma* negative and *Anaplasma* positive sheep in North Iraq.

Hematological parameters	*Anaplasma-*negative sheep	*Anaplasma-*positive sheep
	
	Mean (±SE)	Mean (±SE)
Hb (g/dL)	9.44 (0.14)	6.47 (0.21)**
PCV (%)	31.26 (2.36)	24.40 (3.40)**
RBC (×10^6^/µL)	6.3 (0.12)	4.50 (0.12)**
WBC (×10^3^/µL)	2.69 (1.29)	4.75 (1.21)**
ESR (mm/h)	1.66 (0.12)	6.60 (0.32)*

** Highly significant (p<0.01),

* Significant (p<0.05). SE=Standard error, PCV=Packed cell volume, RBC=Red blood cell, WBC=White blood cell, ESR=Erythrocyte sedimentation rate, SE=Standard error

Table-3 shows the biochemical results between *Anaplasma*-positive and -negative sheep. The mean total bilirubin and the mean BUN levels were significantly higher in *Anaplasma*-positive sheep than in *Anaplasma*-negative sheep. In contrast, the mean total protein, mean albumin, mean globulin, and mean calcium levels were significantly lower in *Anaplasma*-positive sheep than in *Anaplasma*-negative sheep.

**Table-3:** Differences in the mean biochemical parameters between *Anaplasma*-negative and *Anaplasma-*positive sheep in North Iraq.

Biochemical parameters	*Anaplasma-*negative sheep	*Anaplasma-*positive sheep
	
	Mean (±SE)	Mean (±SE)
Total bilirubin (mg/dL)	0.25 (0.14)	0.74 (0.18)**
BUN (mg/dL)	30.90 (6.24)	63.80 (8.20)**
Total protein (g/dL)	7.76 (0.54)	4.87 (0.41)**
Albumin (g/dL)	4.52 (0.28)	2.91 (0.39)*
Globulin (g/dL)	3.25 (0.36)	1.93 (0.32)*
Glucose (mg/dL)	86.25 (1.82)	65.00 (1.45)
Calcium (mg/dL)	11.91 (1.03)	7.85 (1.01)**

** Highly significant (p<0.01),

* Significant (p<0.05). SE=Standard error, BUN=Blood urea nitrogen

## Discussion

There is a scarcity of studies in most parts of the world on the clinical signs associated with *Anaplasma* infection in indigenous sheep and its hematological and biochemical implications. Furthermore, limited information is available regarding the prevalence of this parasite among sheep in Iraq. To the best of our knowledge, our study could be the first to examine the clinical signs and hematological and biochemical parameters associated with ovine anaplasmosis in indigenous sheep in Iraq.

*Anaplasma* species are ubiquitous rickettsial organisms that have been reported in all the six continents of the world [12], especially in the tropics and subtropics, due to a large number of its tick vectors [13].

A lower incidence of *Anaplasma* infection has been reported in Nigeria [12] and Ethiopia [14], with prevalence rates of 5.0% and 6.0%, respectively, documented among sheep. Similar to our finding, an earlier study [9] documented 62.6% prevalence of *A*. *ovis* among sheep in the Kurdistan Region of Iraq. On the basis of our finding and the earlier result [9], we postulate that *Anaplasma* infection is endemic among sheep in Iraq and hence a significant concern to the livestock sector of the country.

In the present study, female sheep were found to be more susceptible to *Anaplasma* infection than male sheep. Similarly, two previous studies conducted in Tunisia and Pakistan, respectively [8,15], also reported a higher prevalence of *Anaplasma* infection among ewes than among rams. The higher prevalence recorded in females may be attributed to extended breeding (lambing and lactation) as well as the stress of breeding, milking, and repeated hormonal changes associated with pregnancy and lambing processes [16,17].

The clinical signs we observed among *Anaplasma*-positive sheep are not peculiar as these signs have been documented among sheep infected with rickettsial organisms [1,4,18,19]. The pale mucous membrane observed in our study may be due to anemia and decreased erythrocyte count and hemoglobin concentration [20], and the clinical sign of jaundice may be due to the increase in total bilirubin [21]. The presence of hemoglobinuria may be attributed to the high rate of intravascular hemolysis resulting in the release of free bilirubin in the urine [19].

Regarding the hematological parameters, we detected a relatively significant reduction in the mean values of total RBC counts, hemoglobin concentration, and PCV in *Anaplasma*-positive sheep, which is consistent with the previous studies conducted on sheep [4,20,22]. This reduction might be due to the intravascular hemolysis of erythrocytes, increased erythrocyte phagocytosis by the reticuloendothelial system, and restricted erythropoietic activity in the bone marrow [23]. In agreement with our finding, a previous study [15] reported an increased WBC count in sheep infected with *Anaplasma* compared to that in non-infected sheep. The relative increase in the total WBC count and the ESR is an indication that the animals were harboring the organism in their system. The increase in ESR values indicates the correlation between the sedimentation of RBCs and the intensity of anemia [21].

The significant increase in the levels of mean serum total bilirubin and mean total BUN that we observed in our study disagrees with a previous study [19], in which sheep were experimentally infected with *A*. *ovis*. In contrast to their finding, we observed a decrease in the mean total protein, mean albumin, and mean globulin levels in *Anaplasma*-infected sheep compared with non-infected sheep. However, a decrease in serum protein, albumin, and globulin levels has been reported in cattle with anaplasmosis [1,24]. The significant increase in total bilirubin levels is concurrent with the appearance of RBCs infected with *Anaplasma* [19]. The increase in total bilirubin levels may be attributed to RBC lysis by the reticuloendothelial system and probably liver cell damage [21,24]. It has also been reported that anaplasmosis causes nephrosis, renal ischemia, dehydration, and some cardiac problems [25], which may be due to increased BUN levels. The reduction in total protein, albumin, and globulin levels might be attributed to their decreased production due to the hepatic cell damage caused by the direct and indirect effects of *Anaplasma* species and the loss of appetite (anorexia) that is associated with the disease [26].

## Conclusion

*Anaplasma* infection among sheep in North Iraq is a significant concern considering that 278 of the 420 sampled sheep in our study were positive for the rickettsial organism, which could have a negative impact on the overall productivity of sheep. Female sheep showed a higher prevalence of the rickettsial organism than male sheep. The hematological and biochemical parameters also showed significant differences between *Anaplasma*-infected and non-infected sheep. Therefore, it is important to pay more attention toward managing tick infestation among sheep to reduce the occurrence of this rickettsial disease for a more robust livestock sector of the Iraqi economy.

## Authors’ Contributions

DAA, FFA, and AYJ planned, designed and conducted the study. SDO did the statistical analysis and wrote the manuscript. FIG helped during the manuscript writing and revision. MSA was involved in the entire study. All authors read and approved the final manuscript.

## Competing Interests

The authors declare that they have no competing interests.

## Publisher’s Note

Veterinary World remains neutral with regard to jurisdictional claims in published institutional affiliation.

## References

[1] Taylor M.A, Coop R.L, Wall R.L. (2016). Veterinary Parasitology.

[2] Ybanez A.P, Inokuma H. (2016). *Anaplasma* species of veterinary importance in Japan. Vet. World.

[3] Ola-Fadunsin S.D, Gimba F.I, Abdullah D.A, Sharma R.S.K, Abdullah F.J.F, Sani R.A. (2018). Epidemiology and risk factors associated with *Anaplasma marginale* infection of cattle in Peninsular Malaysia. Parasitol *Int*.

[4] Yasini S.P, Khaki Z, Rahbari S, Kazemi B, Amoli J.S, Gharabaghi A, Jalali S.M. (2012). Hematologic and clinical aspects of experimental ovine anaplasmosis caused by *Anaplasma ovis* in Iran. Iran. J *Parasitol*.

[5] Yousefi A, Rahbari S, Shayan P, Sadeghi-dehkordi Z, Bohanar A. (2017). Molecular detection of *Anaplasma marginale* and *Anaplasma ovis* in sheep and goat in west highland pasture of Iran. Asian Pac *J Trop Biomed*.

[6] Wang Z, Yang J, Niu Q, Brayton K.A, Luo J, Liu G, Yin H, Liu Z. (2017). Identification of *Anaplasma ovis* appendage associated protein (AAAP) for development of an indirect ELISA and its application. Parasit. Vectors.

[7] Shabana I.I, Alhadlag N.M, Zaraket H. (2018). Diagnostic tool of caprine and ovine anaplasmosis:A direct comparative study. BMC Vet *Res*.

[8] Said M.B, Belkahia H, Alberti A, Zobba R, Bousrih M, Yahiaoui M, Daaloul-Jedidi M, Mamlouk A, Gharbi M, Messadi L. (2015). Molecular survey of *Anaplasma* species in small ruminants reveals the presence of novel strains closely related to *A phagocytophilum* in Tunisia. Vector Borne Zoonotic Dis.

[9] Renneker S, Abdo J, Bakheit M.A, Kullmann B, Beyer D, Ahmed J, Seitzer U. (2013). Coinfection of sheep with *Anaplasma Theileria* and *Babesia* species in the Kurdistan region, Iraq. Transbound. Emerg *Dis*.

[10] Meyer D.J, Harvey W.J. (2004). Veterinary Laboratory Medicine.

[11] Thrall M.A, Weiser G, Allison R, Campbell T.W. (2012). Veterinary Hematology and Clinical Chemistry.

[12] Egbe-Nwiyi T.N, Sherrif G.A, Paul B.T. (2018). Prevalence of tick-borne haemoparasitic diseases (TBHDS) and haematological changes in sheep and goats in Maiduguri abattoir. J Vet Med Anim. Health.

[13] Jongejan F, Uilenberg G. (2004). The global importance of ticks. Parasitology.

[14] Mekuria D, Tesfaye W. (2017). Study on prevalence of hemoparasite in small ruminants in and around Sebata town, Oromia regional State, Ethiopia. Int. J. Adv. Res *Biol Sci*.

[15] Nasreen A, Saeed K, Khan A, Niaz S, Akhtar N. (2016). Serodiagnosis and haematological effect of anaplasmosis in goats and sheep of district Mardan, Khyber Pakhtunkhwa, Pakistan. World J *Zool*.

[16] Ademola I.O, Onyiche T.E. (2013). Haemoparasites and haematological parameters of slaughtered ruminants and pigs at Bodija abattoir, Ibadan, Nigeria. Afr *J Biomed Res*.

[17] Ukwueze C.S, Kalu E.J. (2015). Prevalence of haemoparasites in red Sokoto goats slaughtered at Ahiaeke market, Umuahia, Abia State, Nigeria. J Vet Adv.

[18] Stuen S, Grøva L, Granquist E.G, Sandstedt K, Olesen I, Steinshamn H. (2011). A comparative study of clinical manifestations, haematological and serological responses after experimental infection with *Anaplasma phagocytophilum* in two Norwegian sheep breeds. Acta Vet. Scand.

[19] Khaki Z, Yasini S.P, Jalali S.M. (2018). A survey of biochemical and acute phase proteins changes in sheep experimentally infected with *Anaplasma ovis*. Asian Pac J Trop Biomed.

[20] Radostitis O.M, Gay C.C, Blood D.C, Hinchcliff K.W, Constable P.D. (2000). Veterinary Medicine: A Text of the Diseases of Cattle, Horses, Sheep, Pigs and Goats.

[21] Jain N.C (1986). Veterinary Hematology.

[22] Cruz A.C, Gallois M, Fontugne M, Allain E, Denoual M, Moutaillier S, Devillers E, Zientara S, Memmi M, Chauvin A, Agoulon A, Taussat M.V, Chartier C. (2019). Epidemiology and genetic diversity of *Anaplasma ovis* in goats in Corsica, France. Parasit. Vectors.

[23] Khaki Z, Jalali S.M, Kazemi B, Jalali M.R, Yasini S.P. (2015). A study of hematological changes in sheep naturally infected with *Anaplasma* spp and *Theileria ovis* :Molecular diagnosis. Iran. J Vet Med.

[24] Coskun A, Ekici O.D, Guzelbektes H, Aydogdu U, Sen I. (2012). Acute phase proteins, clinical, hematological and biochemical parameters in dairy cows naturally infected with *Anaplasma marginale*. Kafkas *Univ. Vet. Fakult. Derg*.

[25] Bhatia B.B, Pathak K.M.L, Juyal P.D. (2014). Textbook of Veterinary Parasitology.

[26] Scorpio D.G, Choi K.S, Dumler J.S. (2018). *Anaplasma phagocytophilum* related defects in CD8, NKT, and NK lymphocyte cytotoxicity. Front *Immunol*.

